# Prognostic potential of pre-partum blood biochemical and immune variables for postpartum mastitis risk in dairy cows

**DOI:** 10.1186/s12917-020-02314-6

**Published:** 2020-05-14

**Authors:** Ruo-wei Guan, Di-ming Wang, Bei-bei Wang, Lu-yi Jiang, Jian-xin Liu

**Affiliations:** Institute of Dairy Science, College of Animal Sciences, Zhejiang University, Hangzhou, 310058 P. R. China

**Keywords:** Blood biochemical variables, Neutrophil to lymphocyte ratio, Platelet to lymphocyte ratio, Mastitis, Transition cows

## Abstract

**Background:**

Mastitis is the most frequent diseases for transition cows. Identification of potential biomarkers for diagnosis of mastitis is important for its prevention. Thus, this study was conducted to investigate blood variables related to lipid metabolism, oxidative stress and inflammation, and serum variables that are related to health in postpartum cows.

**Results:**

Seventy-six healthy Holstein dairy cows at week 4 before calving were selected to collect blood samples from weeks − 4 to 4 weekly relative to calving, respectively. Milk yield and composition were recorded weekly. According to the cut-off of somatic cell counts (SCC) for diagnosis of mastitis, 33 cows with SCC ≥ 500,000 cells ml^− 1^, 20 cows with 200,000 cells ≤ SCC < 500,000 cells ml^− 1^, and 23 cows with SCC < 200,000 cells ml^− 1^ were defined as high, middle, and low SCC, respectively. Serum concentrations of β-hydroxybutyrate were higher (*P* < 0.01) during all weeks, and non-esterified fatty acids were higher in high SCC than in low SCC cows from weeks − 3 to 2 relative to calving. Higher serum concentrations of superoxide dismutase (*P* < 0.01) and lower malondialdehyde levels (*P* < 0.01) in low SCC than in high SCC cows indicate that the latter suffered from oxidative stress. The difference analysis of the three groups suggested that none of the above-mentioned variables can be used as potential prognostic candidates. On the other hand, high SCC cows exhibited higher blood neutrophil to lymphocyte ratio (NLR, *P* < 0.01) and platelet to lymphocyte ratio (PLR, *P* < 0.01) than low SCC cows, with a higher NLR (*P* < 0.01) in middle SCC than in low SCC cows. The high SCC cows had lower levels of anti-inflammatory factors including IL-10 (*P* = 0.05), but higher levels of proinflammatory factors such as IL-6 (*P* < 0.01), TNF-α (*P* < 0.05), and PSGL-1 (*P* < 0.01) than low SCC cows.

**Conclusions:**

The significantly different NLR and PLR pre-partum between the middle and low SCC cows suggest their prognostic potential for postpartum mastitis risk.

## Background

The periparturient period is a critical stage for lactating dairy cows. During this stage, cows suffer from a series of physiological and metabolic disorders, including reduced feed intake, and enhanced fat mobilization, reflective of changed lipid metabolism analytes such as non-esterified fatty acids (NEFA) and β-hydroxybutyric acid (BHB), leading to enhanced production of reactive oxygen species (ROS) [1]. Many previous studies agree that significant changes in oxidative stress are observed during the transition period [1, 2], with activity of superoxide dismutase (SOD), glutathione peroxidase (GSH-Px), and malondialdehyde (MDA) as the representative analytes for oxidative stress. The impact of oxidative stress during the transition period may be a major underlying factor of inflammatory and immune dysfunction in dairy cattle, further resulting in metabolic diseases such as mastitis during the post-partum stage [3].

Mastitis is a common disease in non-ruminant and ruminant animals and can be triggered by milk accumulation in mammary gland (MG), and invasion of bacteria such as *Staphylococcus aureus*, finally leading to inflammation [4]. The milk somatic cell counts (SCC) is a sensitive biomarker of mammary inflammation [5, 6]. The International Dairy Federation (IDF) has recommended to establish the thresholds as the cut-off of SCC for diagnosis of mastitis [7, 8]. Variations in SCC depend mainly on the recruitment of leukocytes from blood to MG and finally to milk, most often in response to an inflammatory reaction in the mammary tissue caused by the pathogenic bacterial intrusion [9]. The high incidence of mastitis has always been a problem that needs to be solved. Early monitoring for mastitis is an effective way to prevent its development, but information is limited in this area. Previous studies found evidence of a slightly increased overall breast cancer risk in women with a history of mastitis [10]. Cancer-related inflammatory cells can secrete a series of inflammatory mediators, such as cytokines, and promote tumor proliferation, invasion, and metastasis. This inflammatory response can be reflected by some blood indicators, such as blood neutrophil-to-lymphocyte ratio (NLR), and platelet-to-lymphocyte ratio (PLR) [11]. Therefore, both NLR and PLR have been recognized as effective indicators of systemic inflammatory response and have good prognostic value for some malignant diseases [12].

The immune system in the MG is a defense mechanism involving innate and acquired immunity initiated by the MG to resist bacterial invasion [13]. The papillary tube is the physical and first line defense against bacteria [14]. When the bacteria enter into the MG, white blood cells (WBC) such as polymorphonuclear neutrophils (PMN) are activated through cytokines such as interleukin (IL)-6 and tumor necrosis factor (TNF)-α [15], and are aggregated by chemical induction, causing an oxidative burst and phagocytosis of the pathogen [16]. Meanwhile, IL-6, TNF-α, and IL-10 can affect the function of PMN by regulating their apoptosis [17]. Recent studies have shown that PMN require P-selectin glycoprotein ligand-1 (PSGL-1) to activate them on the vascular endothelium. The PMN interact with PSGL-1 on platelets to migrate in blood vessels and exert anti-inflammatory effects [18]. When the pathogen is not killed by the innate immune defense, acquired immunity will be activated to secrete CD4 and CD8, which recognize and inactivate antigen molecules [19].

Metabolic disorders decline the body’s resistance, while pathogen invasion activates the MG immune system. Mammary immunity affects the function and quantity of immune cells, resulting in differences in NLR and PLR. We hypothesized that cows with high SCC will have alterations in their blood and serum variables before calving, increasing the risk of postpartum mastitis. The objective of the current study was to investigate blood and serum biochemical variables in the transition cows, and to evaluate the prognostic potential of prepartum variables for postpartum mastitis risk.

## Results

### Milk yield and composition

The SCC average from 4 weeks for each cow was used to classify each cow into different SCC groups. According to the cut-off of SCC for diagnosis of mastitis, 33 cows with SCC ≥ 500,000 cells ml^− 1^, 20 cows with 200,000 cells ≤ SCC < 500,000 cells ml^− 1^, and 23 cows with SCC < 200,000 cells ml^− 1^ were defined as high (HSCC), middle (MSCC), and low SCC (LSCC), respectively. These classifications were not based on mastitic signs, but are reflective of mastitis risk.

Milk yield was lower (*P* < 0.01), but milk protein content was higher (*P* = 0.02) in cows with HSCC than in LSCC cows (Table 1). Content of milk urea nitrogen was lower in the HSCC cows than in LSCC cows (*P* < 0.01). The average SCC was 1460.5, 346.0, and 77.6 × 10^3^ cells ml^− 1^ in the HSCC, MSCC, and LSCC cows, respectively.

**Table 1:** Basic information and lactation performance of dairy cows with low, middle, and high somatic cell counts (SCC)

Items	Groups^1^			SEM	*P*-value^2^		
	LSCC	MSCC	HSCC		Hs	Wk	Hs*Wk
Number of cows (head)	23	20	33	–	–	–	–
Parity	2.8	2.9	2.7	0.17	0.26	–	–
SCC (10^3^/mL)	77.6	346.0	1460.5	–	–	–	–
Milk yield (kg)	34.3^a^	31.4^b^	26.5^b^	0.71	< 0.01	< 0.01	0.96
Milk composition (%)							
Milk fat	4.85	5.07	4.88	0.11	0.36	< 0.01	0.53
Milk protein	3.24^b^	3.26^b^	3.35^a^	0.03	0.02	< 0.01	0.48
Milk lactose	4.96^b^	4.97^a^	4.80^b^	0.03	< 0.01	< 0.01	0.75
Total solids	13.2	13.4	13.2	0.12	0.36	< 0.01	0.72
Milk urea nitrogen (mgN/dL)	12.2^a^	10.4^b^	8.5^b^	0.52	< 0.01	0.43	0.95

^1^LSCC = cows with SCC (SCC < 200,000 cells ml^− 1^, *n* = 23); MSCC = cows with middle SCC (200,000 cells ≤ SCC < 500,000 cells ml^− 1^, *n* = 20); HSCC = cows with high SCC (SCC ≥ 500,000 cells ml^− 1^, *n* = 33)

^2^Effect of health status (Hs), sampling week (Wk), and interaction of health status by sampling week (Hs × Wk)

^a,b^Means within the same row followed by different superscripts differ at *P* < 0.05

### Lipid metabolism and oxidative stress Analytes

**Fig. 1:**
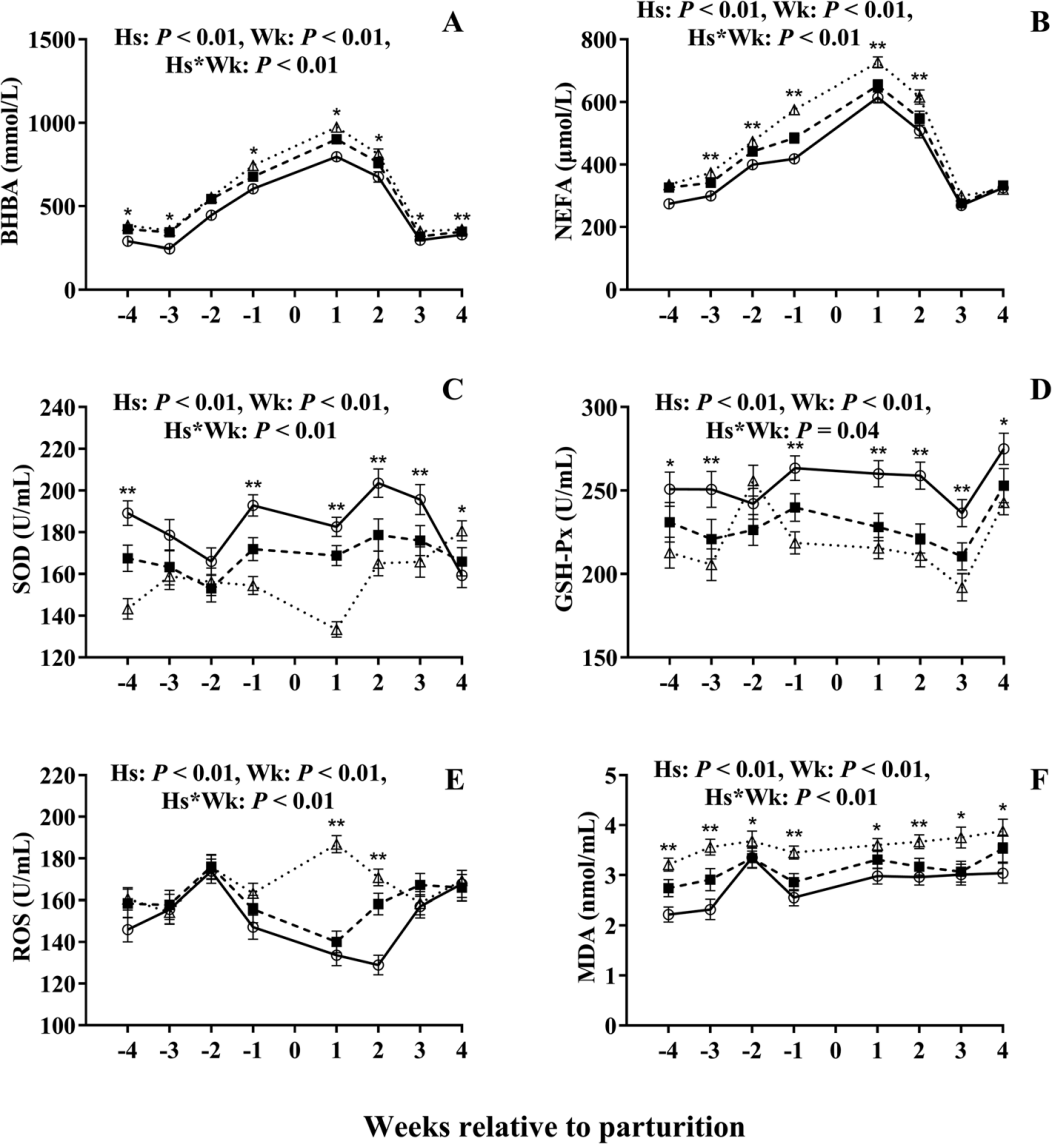
Changes of lipid metabolism and oxidative stress analytes in dairy cows with different somatic cell counts (SCC). -∆-, high SCC (SCC ≥ 500,000 cells ml^− 1^, *n* = 33); -■-, middle SCC (200,000 cells ≤ SCC < 500,000 cells ml^− 1^, *n* = 20); -○-, low SCC (SCC < 200,000 cells ml^− 1^, *n* = 23). BHBA, β-hydroxybutyrate; GSH-Px, glutathione peroxidase; MDA, malondialdehyde; NEFA, non-esterified fatty acid; ROS, reactive oxygen species; SOD, superoxide dismutase. Hs = effect of health status; Wk = effect of sampling week; Hs * Wk = interaction effect (Hs × Wk). * Hs (*P* < 0.05) among three groups; **Hs (*P* < 0.01) among three groups. Error bars indicate SEM

During the whole experimental period, HSCC cows had higher (*P* < 0.01, Fig. 1a) serum concentrations of β-hydroxybutyric acid (BHBA) than LSCC cows, with an interaction effect (*P* < 0.01) of health status and week. Serum concentration of non-esterified fatty acid (NEFA) in HSCC cows was greater (*P* < 0.01, Fig. 1b) than in LSCC cows at all the sampling weeks, except week 4. The MSCC cows had higher (*P* < 0.01) BHBA concentrations than the LSCC cows did at weeks − 4, − 3 and week 1.

Cows with HSCC had lower activity of SOD (*P* < 0.01, Fig. 1c) and GSH-Px (*P* < 0.01, Fig. 1d) compared with LSCC cows during the whole period, except week − 2. The HSCC cows had greater ROS (*P* < 0.01, Fig. 1e) at weeks 1 and 2, and greater activity of MDA (*P* < 0.01, Fig. 1f) during the whole period, than LSCC cows. The significant effects of interaction between health status and week were found (*P* < 0.01, Fig. 1) on serum SOD, GSH-Px, ROS, and MDA.

### Platelets and WBC quantification

**Fig. 2:**
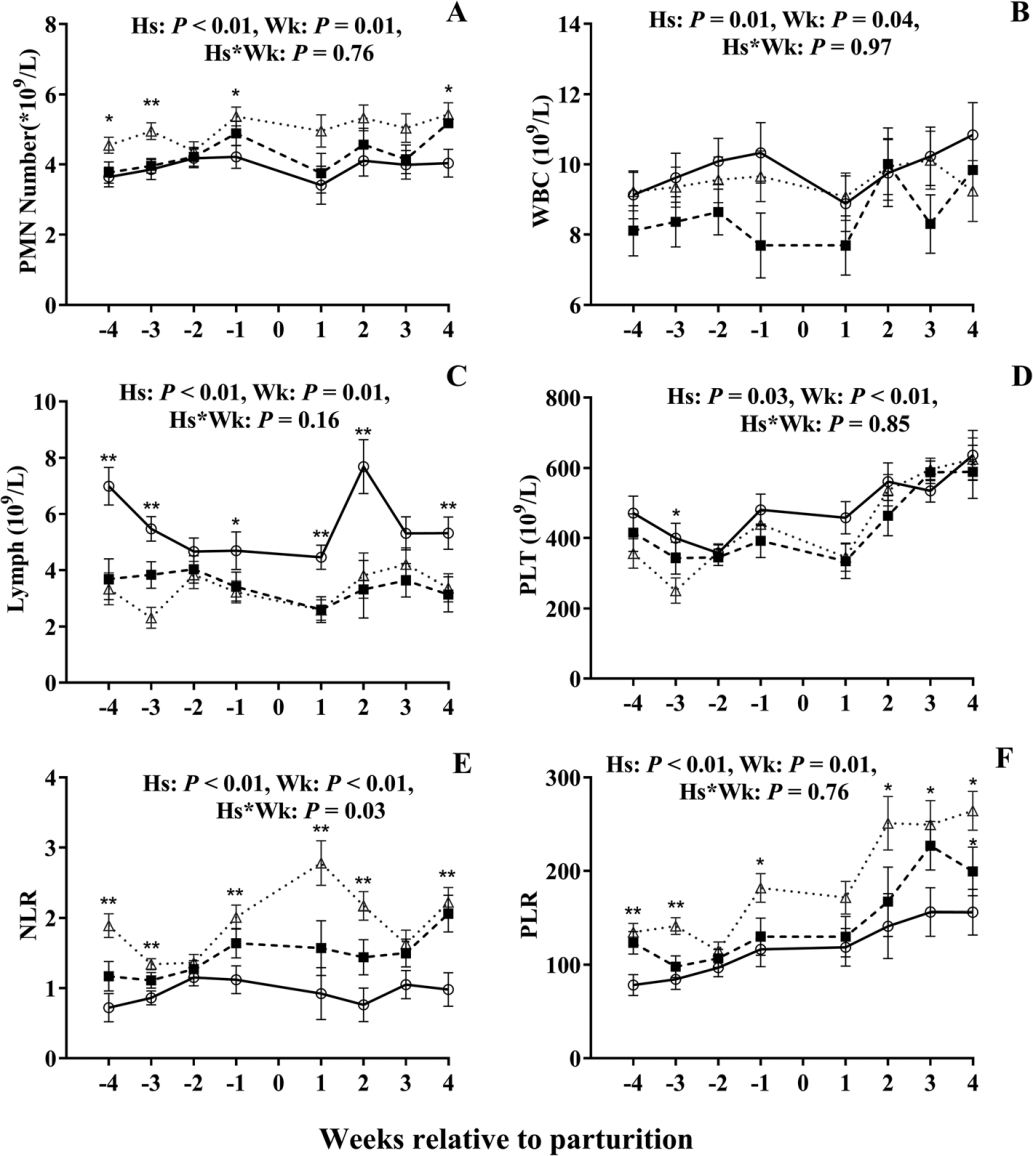
Changes of platelets and white blood cells relative to health in dairy cows with different somatic cell counts (SCC). -∆-, high SCC (SCC ≥ 500,000 cells ml^− 1^, *n* = 33); -■-, middle SCC (200,000 cells ≤ SCC < 500,000 cells ml^− 1^, *n* = 20); -○-, low SCC (SCC < 200,000 cells ml^− 1^, *n* = 23). Lymph, lymphocytes; PMN, polymorphonuclear neutrophils; PLT, platelets; WBC, white blood cells; NLR, neutrophil-to-lymphocyte ratio; PLR, platelet-to-lymphocyte ratio. Hs = effect of health status; Wk = effect of sampling week; Hs * Wk = interaction effect (Hs × Wk). * Hs (*P* < 0.05) among three groups; **Hs (*P* < 0.01) among three groups. Error bars indicate SEM

Numbers of lymphocytes was lower (*P* < 0.01, Fig. 2c) in cows with HSCC than in LSCC cows with lower number of lymphocytes (*P* < 0.01) at weeks − 4 and − 3 in MSCC cow than in LSCC cows, while the PMN, NLR and PLR were greater (*P* < 0.01, Fig. 2a, e, f) in HSCC cows than in LSCC cows during the whole period. The number of platelets was lower (*P* < 0.01, Fig. 2d) in HSCC than in LSCC group until week 2. The cows with HSCC had a lower number of total WBC throughout the study (*P* = 0.01, Fig. 2b) than LSCC cows. The NLR had an effect of interaction between health status and week (*P* = 0.03, Fig. 2e). The MSCC cows had lower NLR (*P* < 0.05) at week 4 than the LSCC cows. At week − 4, cows with MSCC had greater PLR than LSCC cows (*P* < 0.01, Fig. 2f).

### Serum inflammatory cytokines and CD4 and CD8

**Fig. 3:**
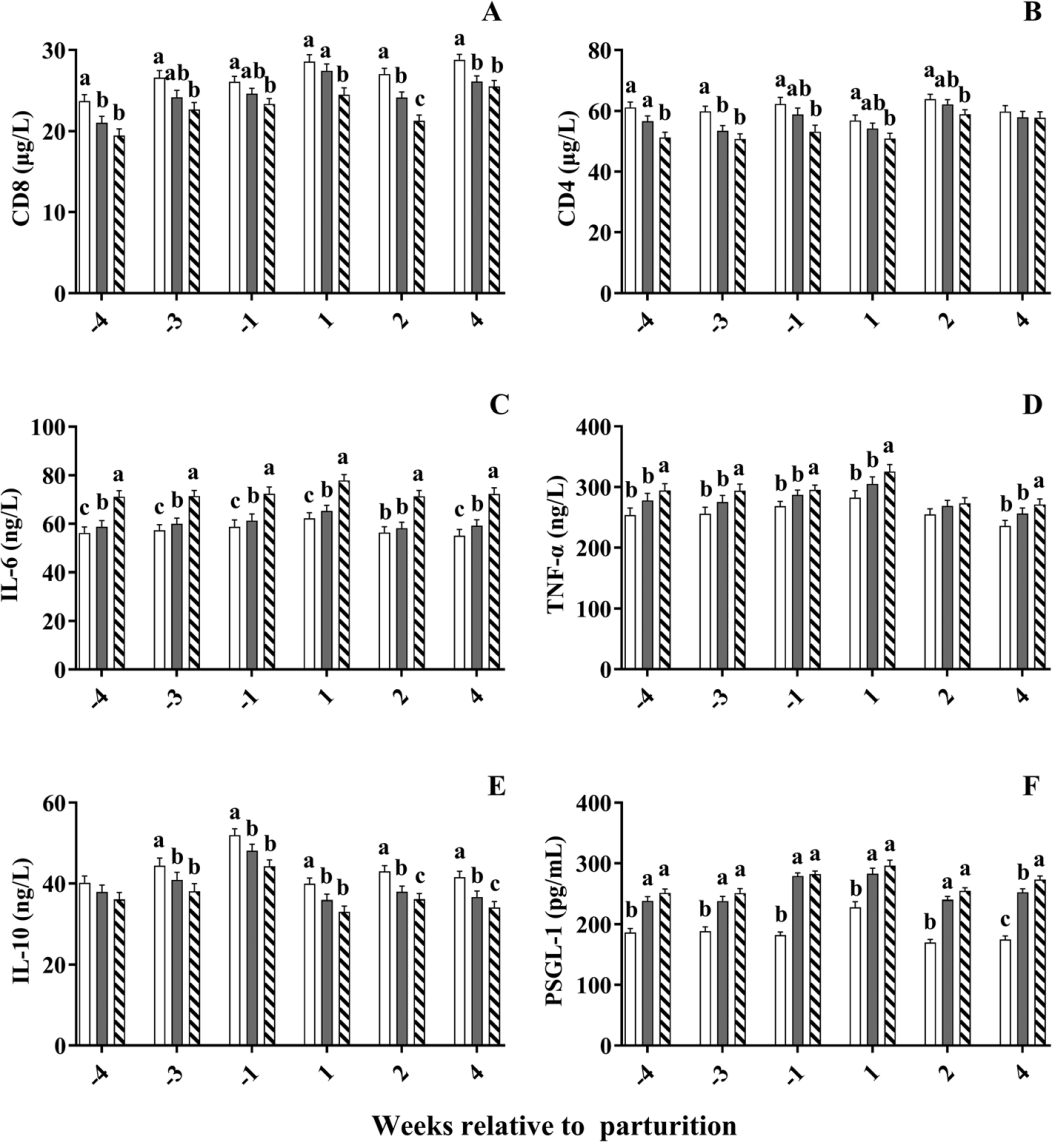
Changes of Serum inflammatory Cytokines and CD4 and CD8 in dairy cows with different somatic cell counts (SCC). , high SCC (SCC ≥ 500,000 cells ml^− 1^, *n* = 33); , middle SCC (200,000 cells ≤ SCC < 500,000 cells ml^− 1^, *n* = 20); , low SCC (SCC < 200,000 cells ml^− 1^, *n* = 23). IL, interleukin; TNF, tumor necrosis factor; PSGL-1, p-selectin glycoprotein ligand-1. Error bars indicate SEM. a, b, c: Different letters within the same week differ (*P* < 0.05)

The content of IL-6 was higher (*P* < 0.01, Fig. 3c) in the HSCC cows during the whole period, with higher (*P* < 0.05) content of IL-6 in the MSCC cows than in LSCC cows at week − 4, − 3, 1, 1 and week 4. The HSCC cows also had higher (*P* < 0.05, Fig. 3d) TNF-α at week − 4, − 3, − 1, 1, and 4 than the MSCC and LSCC cows, but no difference (*P* > 0.05) observed between the two groups. The content of IL-10 was lower (*P* < 0.05, Fig. 3e) in HSCC and MSCC cows than in LSCC cows at week − 3, − 1, 1, 2, and 4; and lower (*P* = 0.03) in HSCC cows than in MSCC cows at week 2 and 4. Serum PSGL-1 content was higher (*P* < 0.01, Fig. 3f) in HSCC and MSCC cows than in LSCC cows during the whole experimental period, with no difference (*P* > 0.05) between the HSCC and MSCC cows except week 4.

Concentration of CD8 was lower in HSCC cows than in LSCC cows (*P* < 0.01, Fig. 3a), with lower values in MSCC cows than in LSCC cows at week 1 (*P* = 0.03) and 2 (*P* = 0.01) postpartum. Serum CD4 concentration was lower in HSCC cows than in LSCC cows during the whole period except week 4 (*P* < 0.01, Fig. 3b), with higher value at week − 4 in MSCC cows than in HSCC cows (*P* < 0.01).

### Correlation analysis and ROC curves

**Fig. 4:**
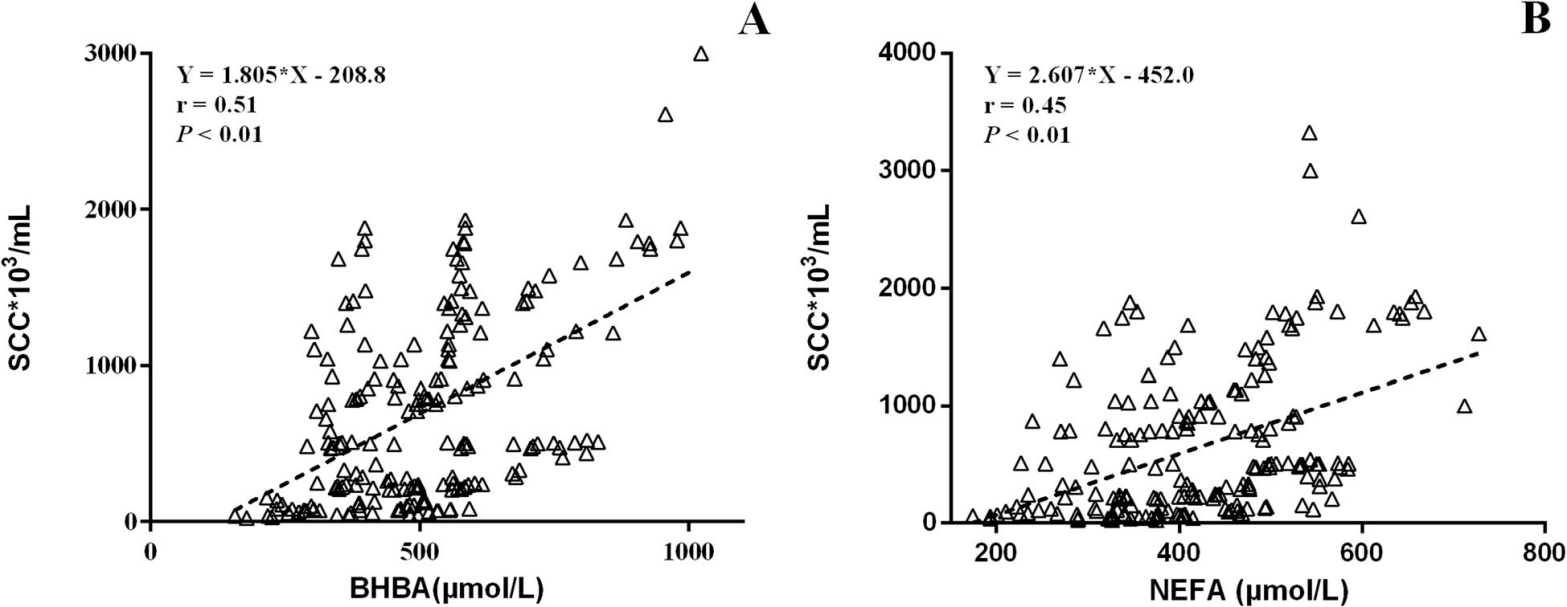
Correlation between somatic cell count (SCC) and prepartum serum concentrations of β-hydroxybutyric acid (BHBA, **a**) or non-esterified acid (NEFA, **b**) in dairy cows (*n* = 76)

Correlations between serum BHBA or NEFA levels in prepartum and milk SCC are shown in Fig. 4. Overall, milk SCC were positively correlated with pre-partum serum BHBA (*r* = 0.51, *P* < 0.01, Fig. 4a) and serum NEFA (*r* = 0.45, *P* < 0.01, Fig. 4b), respectively. Serum BHBA concentration was positively correlated to SCC, with coefficients of 0.65 (week − 4, Figure S1a), 0.73 (week − 3, Figure S1b) and 0.72 (week − 1, Figure S1c). The coefficients of correlation between serum NEFA and SCC in cows were 0.75 (week − 4, Figure S2a), 0.72 (week − 3, Figure S2b), and 0.75 (week − 1, Figure S2c).

**Fig. 5:**
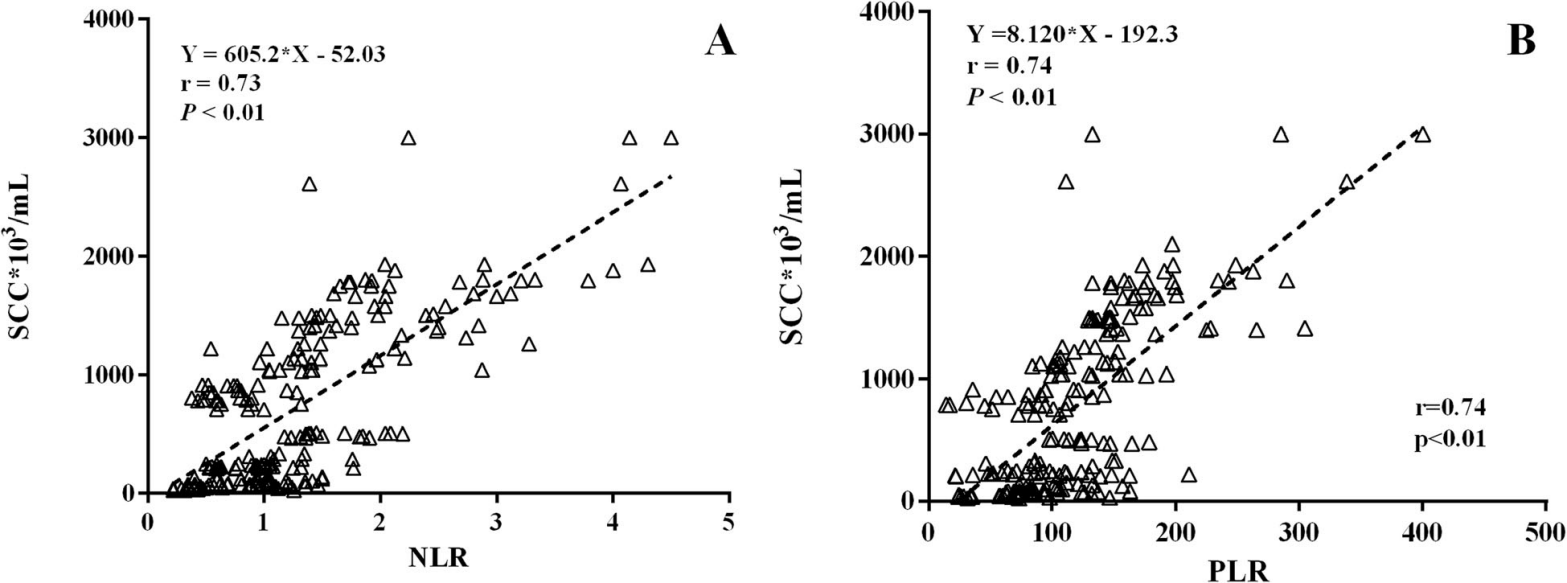
Correlation between somatic cell count (SCC) and prepartum neutrophil-to-lymphocyte ratio (NLR, **a**) or platelet-to-lymphocyte ratio (PLR, **b**) in dairy cows (*n* = 76)

Overall, milk SCC were positively associated with the NLR (*r* = 0.73, *P* < 0.01, Fig. 5a) and PLR (*r* = 0.74, *P* < 0.01, Fig. 5b) before calving. Milk SCC had positive correlations with NLR (*P* < 0.01) at week − 4 (*r* = 0.81, Figure S3a), − 3 (0.79, Figure S3b), and − 1 (*r* = 0.74, Figure S3c), and with the PLR (*P* < 0.01) at week − 4 (*r* = 0.77, Figure S4a), − 3 (*r* = 0.74, Figure S4b), and − 1 (*r* = 0.72, Figure S4c).

The receiver-operator characteristic (ROC) curve of NLR and PLR showed that the area under the curve (AUC) of the NLR in HSCC cows were 0.880 (week − 4, Figure S5a), 0.853 (week − 3, Figure S5b), and 0.883 (week − 1, Figure S5c), and the AUC of the PLR were 0.848 (week − 4, Figure S5a), 0.733 (week − 3, Figure S5b), and 0.765 (week − 1, Figure S5c). The AUC of the NLR in the MSCC cows were 0.765 (week − 4, Figure S6a), 0.625 (week − 3, Figure S6b), and 0.740 (week − 1, Figure S6c), and the AUC of PLR were 0.850 (week − 4, Figure S6a), 0.583 (week − 3, Figure S6b), and 0.635 (week − 1, Figure S6c). In both HSCC and MSCC cows, the AUC of the NLR was higher than that of the PLR. The ROC curve analysis results for BHBA, NEFA (Figure S7, S8), SOD, GSH-Px, ROS, MDA (Figure S9, S10) in HSCC cows and LSCC cows and in MSCC and LSCC cows are shown in the supplementary figures.

## Discussion

Periparturient dairy cows usually suffer from metabolic stress and had increasing serum concentrations of BHBA and NEFA [20]. Cows with higher SCC had higher BHBA and NEFA levels in serum than LSCC-animals in the current study, with the rapidly increased prepartum BHBA levels with time, indicating that perinatal ketosis may have some association with the development of mastitis, consistent with a pervious study in which hepatic dysfunction in pre-partum dairy cows could be associated with post-partum mastitis [21]. Metabolic disorders in dairy cows cause increasing oxidative energy consumption in liver and MG [22]. From our results, HSCC cows had lower activity of SOD and GSH-Px than LSCC cows, indicating greater oxidative stress in HSCC cows [21, 23]. The results of ROC analysis of BHBA and NEFA showed that both had association with the risk in clinical or subclinical mastitis. However, the elevated BHBA and NEFA levels are mainly related to ketosis in dairy cows and were not reflective of perinatal cows experiencing mastitis [24]. Such levels exert relatively minimal alterations to immune function [24] or cannot directly reflect the process of inflammation [25]. Therefore, we did not choose plasma variables (NEFA, BHBA, SOD or GSH-Px) as prognostic candidates for post-partum mastitis.

Severe oxidative stress and high mobilization of body reserves in dairy cows may lead to reduced immune function and increase the risk of postpartum mastitis [25]. It is well known that WBC (PMN, lymphocytes, etc.) and PLT are important immune cells in the blood [26]. However, the difference analysis of the three groups suggested that none of the above-mentioned variables can be used as potential prognostic candidates under the current experimental conditions. The derangement of the WBC and PLT are known as poor independent prognostic factors for many solid tumors [27]. On the other hand, blood NLR has been used as one of the indicators to evaluate the body’s immune system, a strong prognostic factor of certain cancers [13], and as a predictor of inflammatory or infectious lesions [28] and postoperative complications [29]. In addition, blood PLR is a novel inflammatory marker, and has been demonstrated to be a predictor of various cardiovascular diseases and tumors [30], and the risk for critical limb ischemia [31]. Correlation analysis suggested that both prepartum NLR and PLR were positively associated with milk SCC in dairy cows in the current study, suggesting an increased risk of postpartum mastitis in cows with higher NLR and PLR before calving. One possibility is that neither PMN nor WBC alone can be a prognostic index, as there exists a balance between lymphocytes and neutrophils, and a balanced status of inflammatory response [32]. Similarly, a balance exists between platelet and lymphocyte counts in blood. An increased platelet count will accelerate bacterial migration, while a decreased lymphocyte count will reduce the animals’ ability to resist inflammation. A combination of these two factors may be more comprehensive in the status of inflammation incidence [18]. In this study, the relatively lower concentration of CD4 and CD8 in the serum of animals with HSCC indicated their dysfunction in such dairy cows, which further lowered the ability of lymphocytes to resist diseases and increased the risk of postpartum mastitis in dairy cows. This is consistent with the results by Koya et al. [33], who reported reduced CD4 and CD8 T cells in peripheral blood of patients with non-lactation mastitis.

**Fig. 6:**
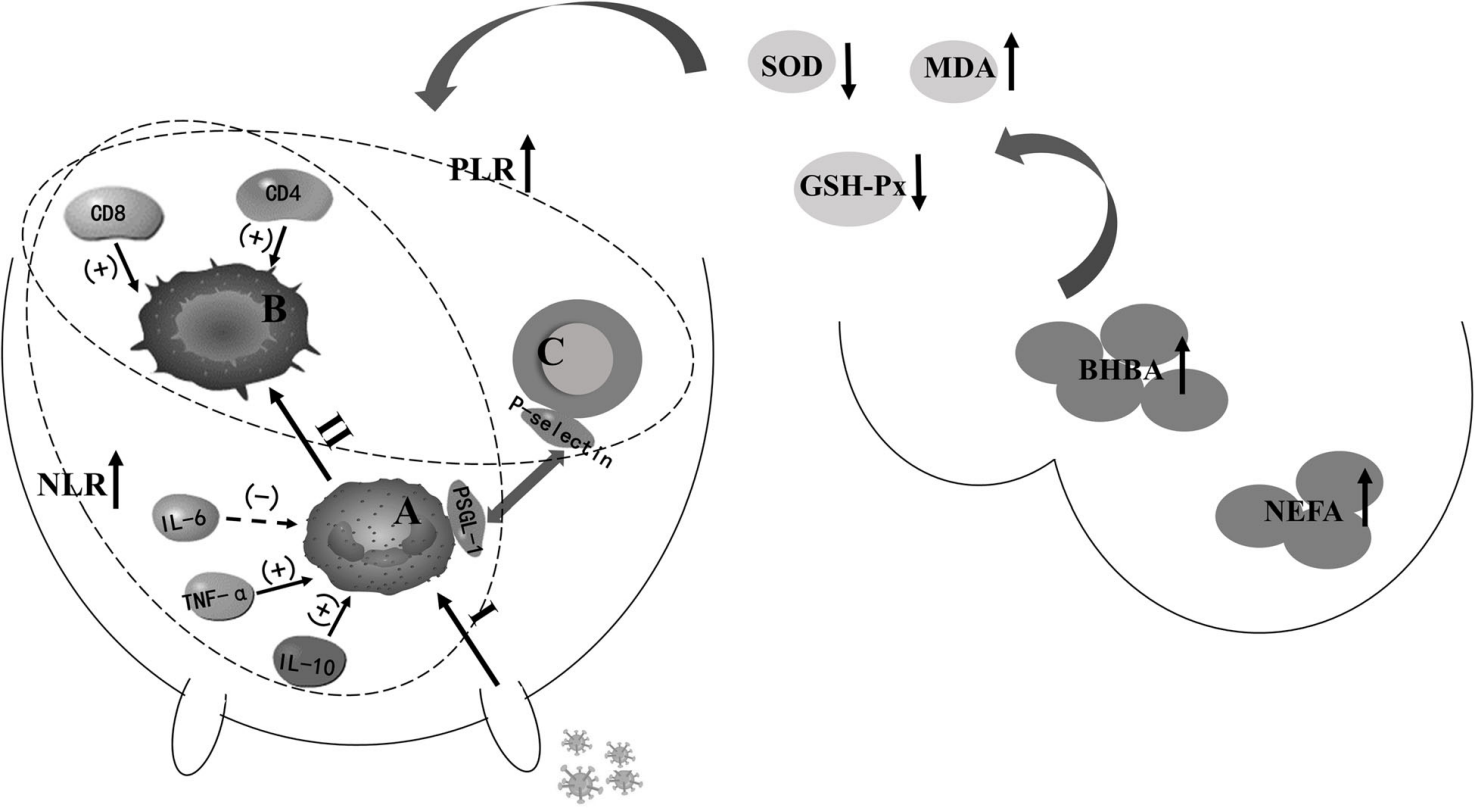
The immune processes in the dairy cows with high somatic cell counts (SCC). Excessive body fat mobilization increases the serum concentrations of β-hydroxybutyric acid (BHBA) and non-esterified acid (NEFA), reduces the activities of serum superoxide dismutase (SOD) and glutathione peroxidase (GSH-Px), and increases the activity of serum malondialdehyde (MDA), leading to oxidative stress and changed immune status of the HSCC cows. The tissue innate immune systems will then be activated, with polymorphonuclear neutrophils (PMN) playing an important role. When the pathogen is not killed by the innate immune defenses, the acquired immune system is activated, with lymphocytes playing an important role in this process. During the whole period, the cytokines such as interleukin (IL)-6, tumor necrosis factor (TNF)-α, IL-10, and p-selectin glycoprotein ligand-1 (PSGL-1) play different roles, and increase blood neutrophil-to-lymphocyte ratio (NLR) and platelet-to-lymphocyte ratio (PLR). (+) indicates promotion, (−) indicates inhibition; I, innate immunity, II, acquired immunity; **a** polymorphonuclear neutrophils; **b** lymphocyte; **c** Platelet

Changes in blood cytokine levels are predominant regulators of blood NLR and PLR alteration in pre-partum dairy cows [13–15]. Both IL-6 and TNF-α are important pro-inflammatory factors that affect PMN formation and release [34]. Cytokine IL-6 inhibits apoptosis of PMN in patients with osteomyelitis [35] and could inhibit the natural apoptosis of endogenous neutrophils [36]. On the other hand, TNF-α promotes the apoptosis of peripheral blood neutrophils in rats [37]. In the present study, the level of IL-6 was significantly different among the three groups, and may inhibit the apoptosis of PMN and thus increase the number of PMN. In general, elevated levels of IL-6 in the serum of pre-partum HSCC cows, compared to those of LSCC cows, would increase the release and activation of PMN, inhibit the apoptosis of PMN, then increase the number of PMN and NLR. The IL-10 can inhibit the synthesis of pro-inflammatory cytokines by T cells and monocytes/macrophages [38]. In the present study, HSCC cows had lower levels of pre-partum serum IL-10 than LSCC cows, leading to decreased inflammation resistance, and an increased postpartum mastitis risk. In addition, IL-10 can promote the apoptosis of PMN [39]. Compared with the LSCC cows, HSCC cows had lower IL-10 activity in blood, and possibly exhibited reduced PMN apoptosis, and increased PMN and NLR levels in blood. It is known that neutrophils interact with PSGL-1 on platelets to migrate in blood vessels and exert anti-inflammatory effects; PSGL-1 knock out mice had reduced numbers of blood neutrophils migrating along the inner surface of blood vessel [18]. The higher levels of PSGL-1 in the HSCC cows in this study suggested that PMN of HSCC cows may be activated to increase adhesion, which further elevates the expression of TNF-α [26]. In summary, excessive lipid mobilization of cows caused higher serum concentration of NEFA and BHBA, and lower SOD, and GSH-Px but higher MDA, resulting in the increased oxidative stress. Excessive oxidative stress would destroy the cow’s immune system. Increased NLR and PLR in HSCC cows indicate that the cows were in an inflammatory status and could be a result of lower IL-10, and higher IL-6, TNF-α, and PSGL-1 (Fig. 6).

From the ROC curve analysis, it is indicated that both per-partum NLR and PLR had good predictive potential in higher SCC in post-partum dairy cows, indicating an increased risk of mammary disease. A higher AUC of the NLR than the PLR suggested that NLR may be an indicator better than the PLR,

According to the prevalence of the disease in the farm evaluated, the sample size is limited in the current study as a preliminary work. In a subsequent study, the sample size could be expanded to validate the results of this study.

## Conclusion

Post-partum cows with HSCC and MSCC had greater body fat mobilization and increased oxidative stress during their pre-partum stage, compared with LSCC cows. According to the ROC curve analysis, NLR and PLR had prognostic potential for postpartum risk in clinical and subclinical mastitis. Our study suggested that pre-partum monitoring blood variables such as NLR and PLR can be an efficient method to predict the incidence of clinical and sub-clinical mastitis risk in post-partum dairy cows, helping dairy producers to treat such cows in advance to prevent mastitis risk.

## Methods

### Animals and management

The experimental procedures used in this study were approved by the Animal Care Committee of Zhejiang University (Hangzhou, China) and were in accordance with the university’s guidelines for animal research. Eighty-six healthy pregnant Holstein dairy cows at 4 weeks before calving were selected at the Hang-Jiang Dairy Farm, Zhejiang Province, China; where a total of 1500 dairy cattle are reared with around 800 lactating cows. Permission to collect sample was obtained from the farm owner. All the selected cows were similar in parity (2.93, SD 0.89) and body condition score (3.29, SD 0.37). Experiment lasted from mid-September to late November, 2017. A veterinarian checked the selected cows before the trial and no disease record was observed. Then, cows were monitored weekly during the whole experiment period to evaluate their health status based on the clinical signs. As a result, ten cows were removed due to their health problems such as clinical mastitis, milk fever, and ketosis. Thus, 76 cows were finally enrolled in this study. Cows were housed in individual tie stalls, bedded with sawdust, milked three times daily, and had free access to water throughout the experiment. The cows were kept as free stall in the barn while the experiment was conducted. Therefore, the animals were released after the study was completed.

Diets were offered as total mixed rations ad libitum three times daily at 0630, 1300, 1830 h to allow approximately 5% orts. All rations were formulated to meet the nutrient requirements of dry and early lactating cows [40]. Ingredient and chemical composition of the diets fed during the prepartum and postpartum periods are shown in Supplementary Table S1.

### Experimental design and sampling

Before the data were collected, three thresholds of SCC were designed according to the IDF recommendations [7, 8]. Blood samples were collected during the whole period from weeks − 4 to 4 relative to calving. From calving to week 4, milk yield was recorded and milk samples were collected for analysis of milk composition including SCC. After all the samples were collected, 76 dairy cows were divided into three groups based on the pre-designed thresholds of the SCC to compare their differences in blood variables and to analyze the correlation between pre-partum blood or serum variables and SCC.

Blood samples were obtained from the coccygeal vein at around 0930 h – 1000 h once a week at weeks − 4, − 3, − 2, − 1, 1, 2, 3, and 4 relative to calving. The blood was collected into 2 mL EDTA anticoagulant vacutainer tubes (EDTA k2), and then sent to an animal hospital (Nargis Animal Hospital, Hangzhou, China) within 2 h for analysis of blood variables. Another blood sample was collected into 5 mL vacutainer tubes (Yuli Medical Instrument Co., Jiangsu, China) and then kept at 4 °C until separation of serum. Clotted blood was centrifuged at 3000×g for 15 min. The serum was transferred to a sterile 1.5 mL RNase-free plastic test tube (MCT-150-C-S, Axygen, Corning, NY, USA), and then stored at − 80 °C until analysis.

Milk yield was recorded daily during the sampling periods, and milk samples were collected once a week at weeks 1, 2, 3, and 4 with milk-sampling devices (Waikato Milking Systems NZ Ltd., Hamilton, New Zealand). Daily samples were composited proportionally (4:3:3 according to three times of milking) and stored with added bronopol tablets (milk preservative; D & F Control System Inc., San Ramon, CA, USA) for later analysis of milk compositions.

### Analysis of blood, serum and milk samples

Number of PMN, lymphocytes, and platelet were quantified using an automatic blood cell analyzer (B2600, Mandray, Shexhen, China). The NLR and PLR were then calculated. An Auto Analyzer 7020 instrument (Hitachi High-technologies Corporation, Tokyo, Japan) was used to determine serum lipid metabolism analytes including NEFA, and BHBA as well as oxidative stress analytes such as ROS, SOD, GSH-Px and MDA, with colorimetric commercial kits (Ningbo Medical System Biotechnology Co., Ltd., Ningbo, China). The serum concentrations of CD4 (Catalog No. MB-6980A), CD8 (MB-4853A), IL-6 (MB-4905A), IL-10 (MB-4931A), TNF-α (MB-4838A), and PSGL-1 (MB-9620A) were analyzed using commercially available ELISA kits specific for the bovine species (Jiangsu Enzyme Biotechnology Co., Ltd., Jiangsu, China). Before the samples were analyzed, an engineer from the instrument was requested to calibrate the instrument with a standard to validate the analytical procedures.

Milk samples were analyzed for milk protein, fat, lactose, total solids, urea nitrogen and SCC using a Combi Foss FT+ instrument (Foss Electric, Hillerød, Denmark).

### Statistical analyses

In order to identify the prognostic factors of clinical and subclinical mastitis in cows, data were compared for LSCC, MSCC and HSCC cows separately at each time point. Data on lactation performance, blood variables related to lipid metabolism, oxidative stress and inflammation, and serum variables were analyzed using the PROC MIXED procedure with repeated measurement (SAS Institute Inc., Cary, NC, USA). The model included the health status (LSC, MSCC, HSCC), sampling weeks and their interaction (health status × wk) as the fixed effects, and cow within the block as a random effect. Spearman correlation analysis was conducted between pre-partum blood or serum variables and SCC in three groups using GraphPad Prism 6 software (GraphPad Software Inc., La Jolla, CA, USA). Significance was declared at *P* < 0.05, and tendency was defined at 0.05 < *P* < 0.10. Graphs were generated with GraphPad Prism 6 software (GraphPad).

Biomarker profiles and quality of the biomarker sets were assessed using ROC curves, as calculated by IBM SPSS Statistics 20.0 software (International Business Machines Corp., Armonk, New York 10,504, USA). The ROC curves are often summarized into a single metric known as the AUC, which is indicative of the accuracy of a test for correctly distinguishing one group from others. The NLR and PLR were evaluated with ROC analysis, and the higher AUC values associated with NLR and PLR were used to determine the most predictive critical threshold for disease identification. Sensitivity was defined as the proportion of animals diagnosed with disease that were above the threshold of a given blood variable, and specificity was the proportion of animals that did not have disease below a given threshold [41]. If all positive samples are ranked before negative ones, the AUC is 1.0, which indicates a perfectly discriminating test.

## Supplementary information

**Additional file 1 Table S1**. Ingredient and chemical composition of the diets fed during the prepartum and postpartum periods. **Figure S1**. Correlation between somatic cell count (SCC) and β-hydroxybutyric acid (BHBA) in dairy cows. **Figure S2.** Correlation between somatic cell count (SCC) and non-esterified acid (NEFA) in dairy cows. **Figure S3.** Correlation between somatic cell count (SCC) and neutrophil-to-lymphocyte ratio (NLR) in dairy cows. **Figure S4.** Correlation between somatic cell count (SCC) and platelet-to-lymphocyte ratio (PLR) in dairy cows. **Figure S5.** Receiver-operator characteristic curves of blood neutrophil-to-lymphocyte ratio (NLR) and blood platelet to-lymphocyte ratio (PLR) in dairy cows with low (LSCC) and high somatic cell count (HSCC). **Figure S6**. Receiver-operator characteristic curves of blood neutrophil-to-lymphocyte ratio (NLR) and blood platelet to-lymphocyte ratio (PLR) in dairy cows with low (LSCC) and middle somatic cell count (MSCC). **Figure S7.** Receiver-operator characteristic curves of lipid metabolism analytes in dairy cows with low (LSCC) and high somatic cell count (HSCC). **Figure S8. R** Receiver-operator characteristic curves of lipid metabolism analytes in dairy cows with low (LSCC) and middle somatic cell count (MSCC). **Figure S9.** Receiver-operator characteristic curves of serum anti-oxidative analytes in dairy cows with low (LSCC) and high somatic cell count (HSCC). **Figure S10.** Receiver-operator characteristic curves of serum anti-oxidative analytes in dairy cows with low (LSCC) and middle somatic cell count (MSCC).

## Data Availability

The datasets used and/or analysed during the current study are available from the corresponding author on reasonable request.

## Abbreviations

AUC: Area under the curve
BHBA: β-hydroxybutyrate
GSH-Px: Glutathione peroxidase
IL: Interleukin
MDA: Malondialdehyde
MG: Mammary gland
NEFA: Non-esterified fatty acid
NLR: Neutrophil to lymphocyte ratio
PLR: Platelet to lymphocyte ratio
PLT: Platelet
PMN: Poly-morphonuclear neutrophils
PSGL-1: P-selectin glycoprotein ligand-1
ROC: Receiver-operator characteristic
ROS: Reactive oxygen species
SCC: Somatic cell counts
SOD: Superoxide dismutase
TNF: Tumor necrosis factor
WBC: White blood cell
