# Supplementary material for: Prognostic potential of pre-partum blood biochemical and immune variables for postpartum mastitis risk in dairy cows
Source: BMC Vet Res. 2020 May 14;16:136. doi: 10.1186/s12917-020-02314-6 (PMC7222453; doi:10.1186/s12917-020-02314-6)
Supplement: Supplementary file 1 — Additional file 1 Table S1. Ingredient and chemical composition of the diets fed during the prepartum and postpartum periods. Figure S1. Correlation between somatic cell count (SCC) and β-hydroxybutyric acid (BHBA) in dairy cows. Figure S2. Correlation between somatic cell count (SCC) and non-esterified acid (NEFA) in dairy cows. Figure S3. Correlation between somatic cell count (SCC) and neutrophil-to-lymphocyte ratio (NLR) in dairy cows. Figure S4. Correlation between somatic cell count (SCC) and platelet-to-lymphocyte ratio (PLR) in dairy cows. Figure S5. Receiver-operator characteristic curves of blood neutrophil-to-lymphocyte ratio (NLR) and blood platelet to-lymphocyte ratio (PLR) in dairy cows with low (LSCC) and high somatic cell count (HSCC). Figure S6. Receiver-operator characteristic curves of blood neutrophil-to-lymphocyte ratio (NLR) and blood platelet to-lymphocyte ratio (PLR) in dairy cows with low (LSCC) and middle somatic cell count (MSCC). Figure S7. Receiver-operator characteristic curves of lipid metabolism analytes in dairy cows with low (LSCC) and high somatic cell count (HSCC). Figure S8. R Receiver-operator characteristic curves of lipid metabolism analytes in dairy cows with low (LSCC) and middle somatic cell count (MSCC). Figure S9. Receiver-operator characteristic curves of serum anti-oxidative analytes in dairy cows with low (LSCC) and high somatic cell count (HSCC). Figure S10. Receiver-operator characteristic curves of serum anti-oxidative analytes in dairy cows with low (LSCC) and middle somatic cell count (MSCC). [file 12917_2020_2314_MOESM1_ESM.docx]

**Supplementary materials**

Table S1. Ingredient and chemical composition of the diets fed during the prepartum and postpartum periods

| Item | Prepartum | Postpartum | |
| --- | --- | --- | --- |
| Ingredients, % of DM |  | |  |
| Corn grain, ground | 12.41 | | 13.39 |
| Steam-flaked corn | 7.15 | | 11.93 |
| Soybean meal | 8.64 | | 13.68 |
| Wheat bran | 5.30 | | 0.00 |
| Beet pulp | 6.70 | | 9.26 |
| Corn silage | 25.39 | | 21.05 |
| Alfalfa | 6.78 | | 16.86 |
| Oat Hay | 18.08 | | 5.62 |
| Sodium bicarbonate | 0.40 | | 0.72 |
| Calcium hydrophosphate | 0.40 | | 0.48 |
| Limestone, ground | 0.60 | | 0.66 |
| Fatty acid calcium | 0.00 | | 0.76 |
| Salt | 0.39 | | 0.46 |
| Premix^1^ | 0.37 | | 0.44 |
| Mycotoxin binder | 0.05 | | 0.07 |
| Active yeast | 0.00 | | 0.07 |
| Brewer’s grains | 7.34 | | 4.55 |
| Composition, % of DM |  | |  |
| Crude protein | 11.0 | | 17.5 |
| Neutral detergent fiber | 48.5 | | 37.1 |
| Acid detergent fiber | 27.6 | | 20.8 |
| Crude ash | 8.0 | | 7.8 |
| Calcium | 0.60 | | 1.10 |
| Phosphorus | 0.40 | | 0.41 |
| Net energy for lactation, Mcal / kg DM | 1.38 | | 1.63 |

^1^ Formulated to contain (per kilogram of premix) 220 to 400 KIU of vitamin A, 50 to 100 KIU of vitamin D3, ≥2250 IU of vitamin E, ≥40 mg of D-Biotin, ≥380 mg of niacinamide, ≥40 mg of beta-carotene, 0.2 to 0.7 g of Cu, 1.0 to 3.8 g of Zn, 0.8 to 3.0 g of Mn, 12.5 to 100 mg of I, 8.0 to 25 mg of Se, 2.5 to 50 of mg Co, 10.0 to 30.0% of Ca, 10.0 to 30.0% of NaCl, and ≥1.5% of total phosphorus, and ≤ 10% water.

**
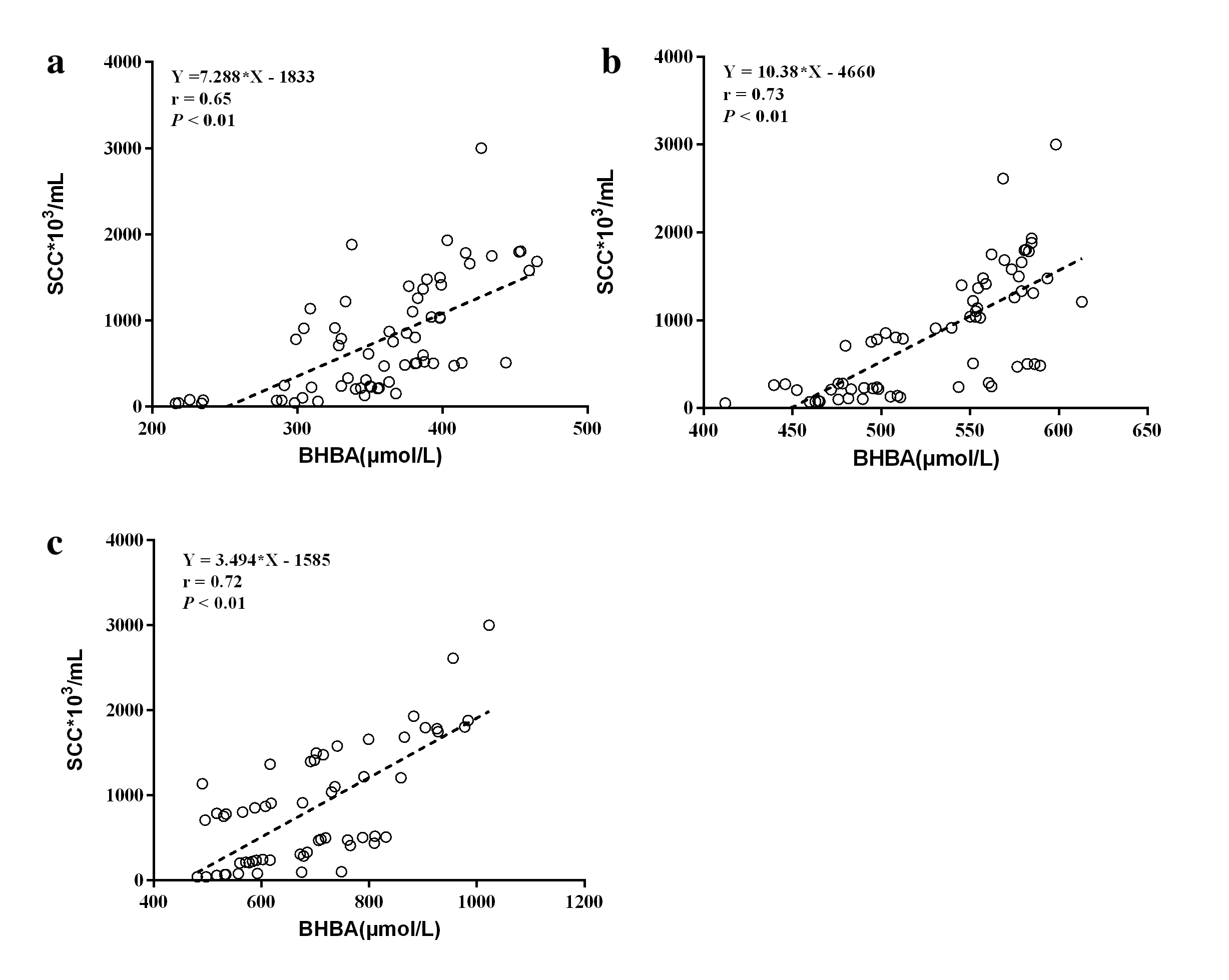
**

**Figure S1**. Correlation between somatic cell count (SCC) and β-hydroxybutyric acid (BHBA) in dairy cows at -4w (a), -3w (b), and -1w (c) relative to calving. N = 76


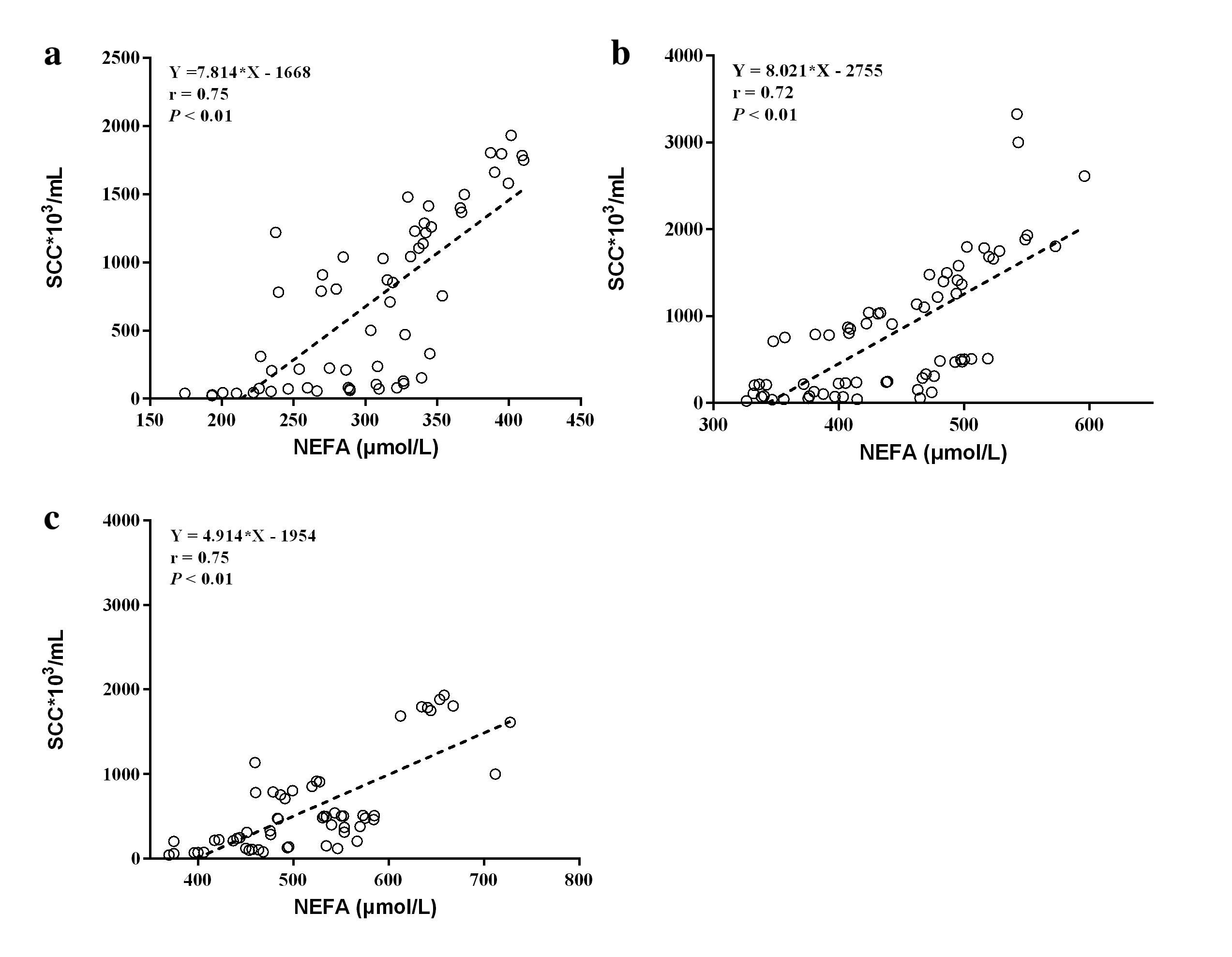


**Figure S2.** Correlation between somatic cell count (SCC) and non-esterified acid (NEFA) in dairy cows at -4w (a), -3w (b), and -1w (c) relative to calving. N = 76


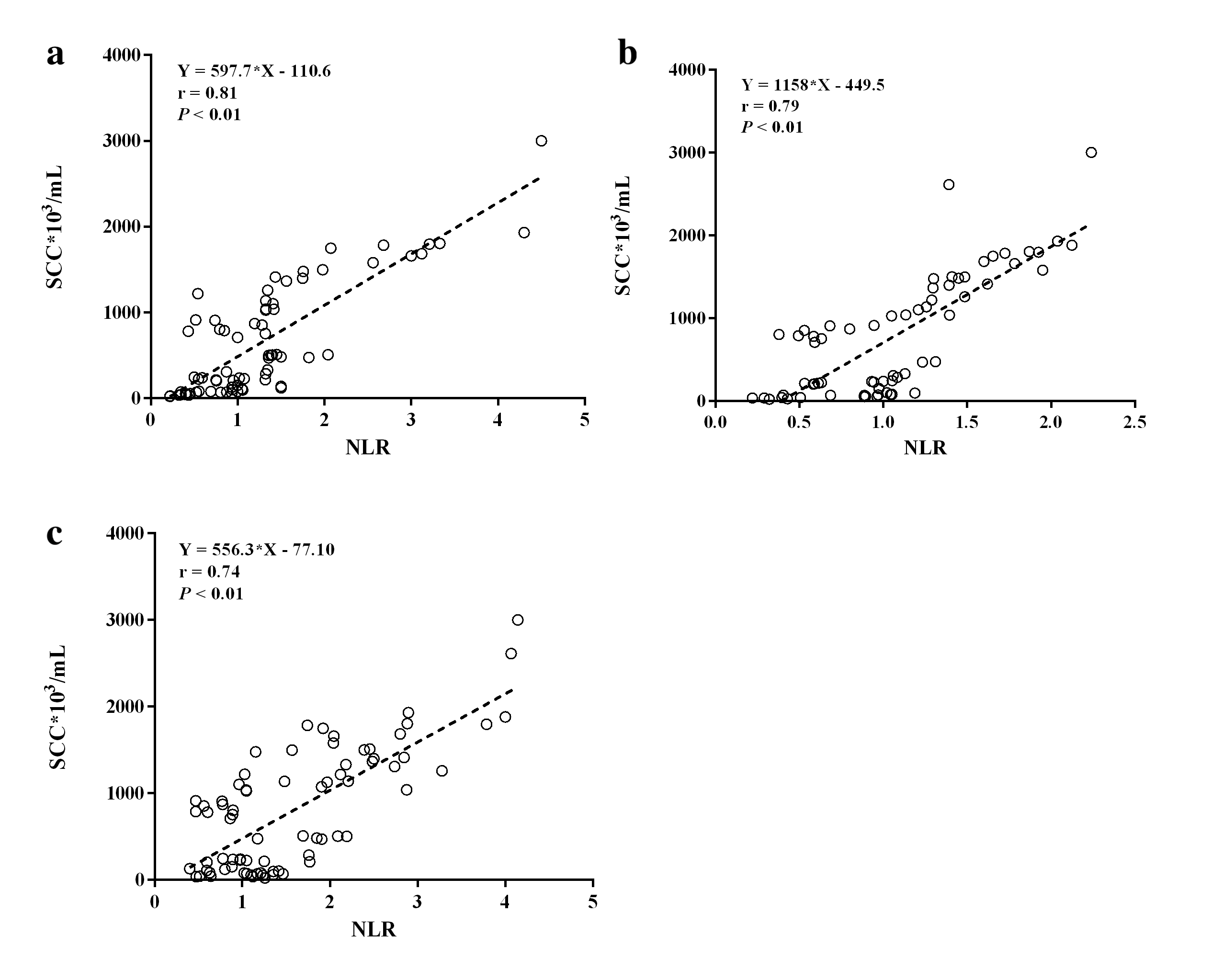


**Figure S3.** Correlation between somatic cell count (SCC) and neutrophil-to-lymphocyte ratio (NLR) in dairy cows at -4w (a), -3w (b), and -1w (c) relative to calving. N = 76


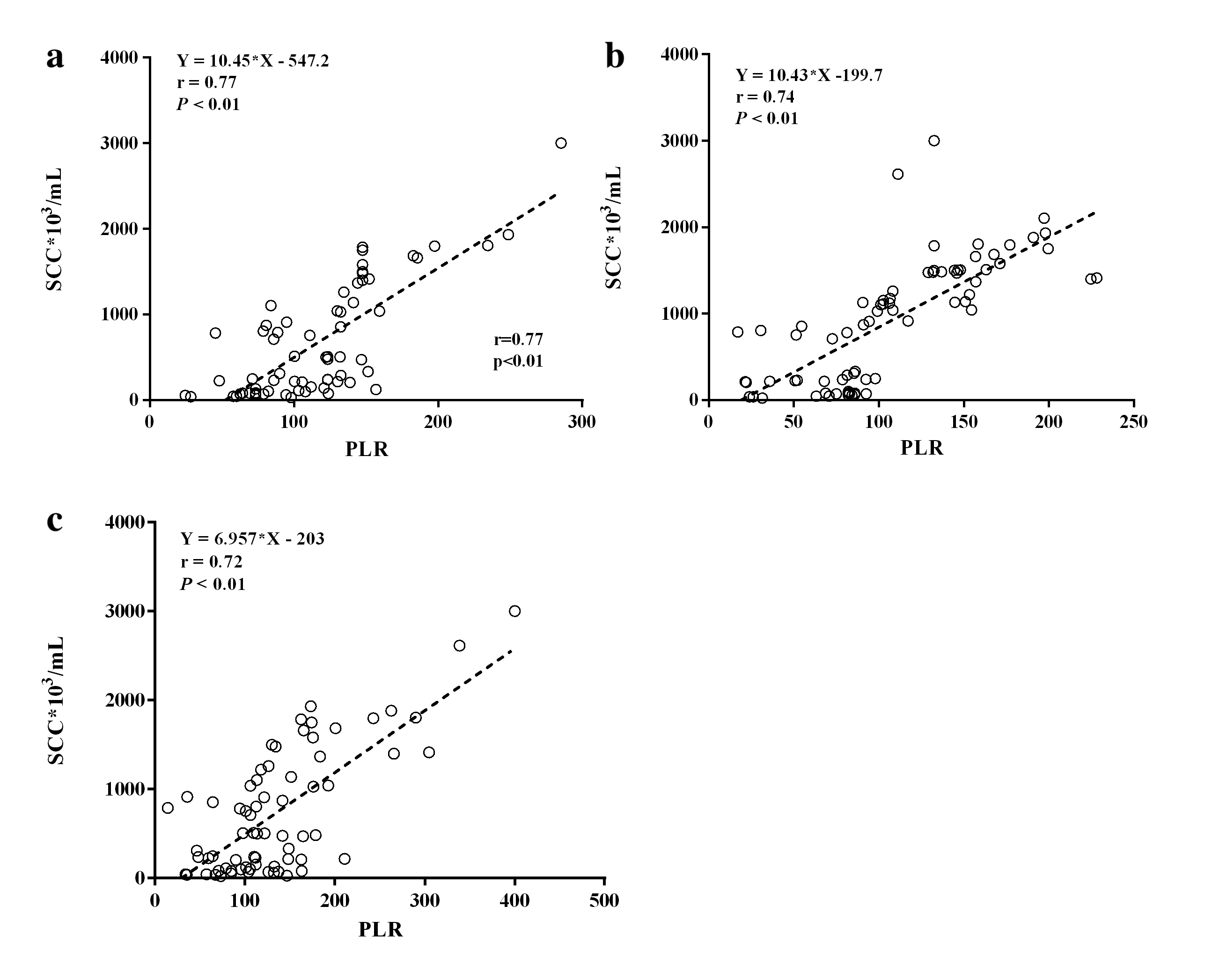


**Figure S4.** Correlation between somatic cell count (SCC) and platelet-to-lymphocyte ratio (PLR) in dairy cows at -4w (a), -3w (b), and -1w (c) relative to calving. N = 76


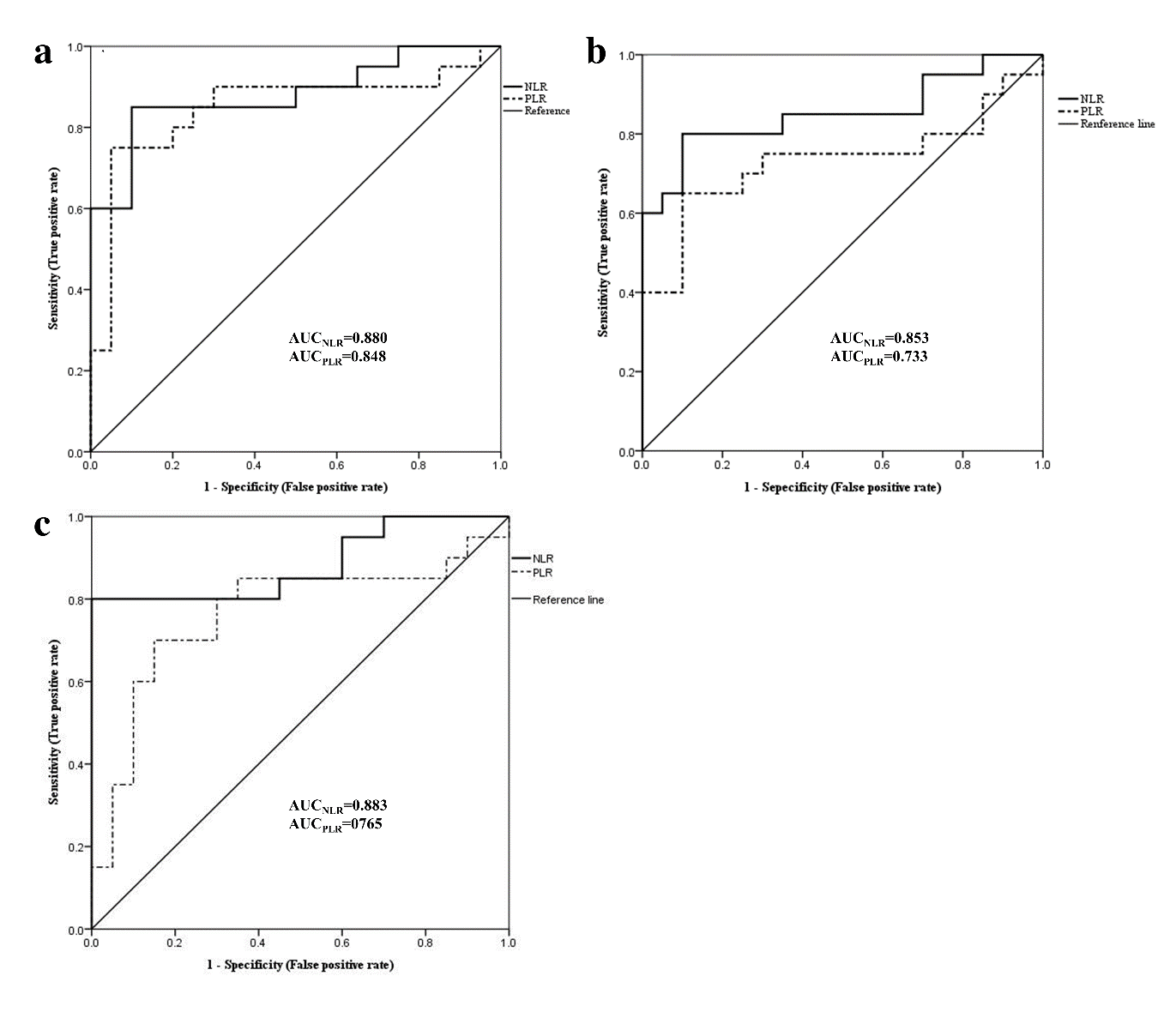


**Figure S5.** Receiver-operator characteristic curves of blood neutrophil-to-lymphocyte ratio (NLR) and blood platelet to-lymphocyte ratio (PLR) in dairy cows with low (LSCC) and high somatic cell count (HSCC) at -4w (a), -3w (b), and -1w (c) relative to calving. N_(LSCC)_=23, N_(HSCC)_=33; AUC, area under the curve


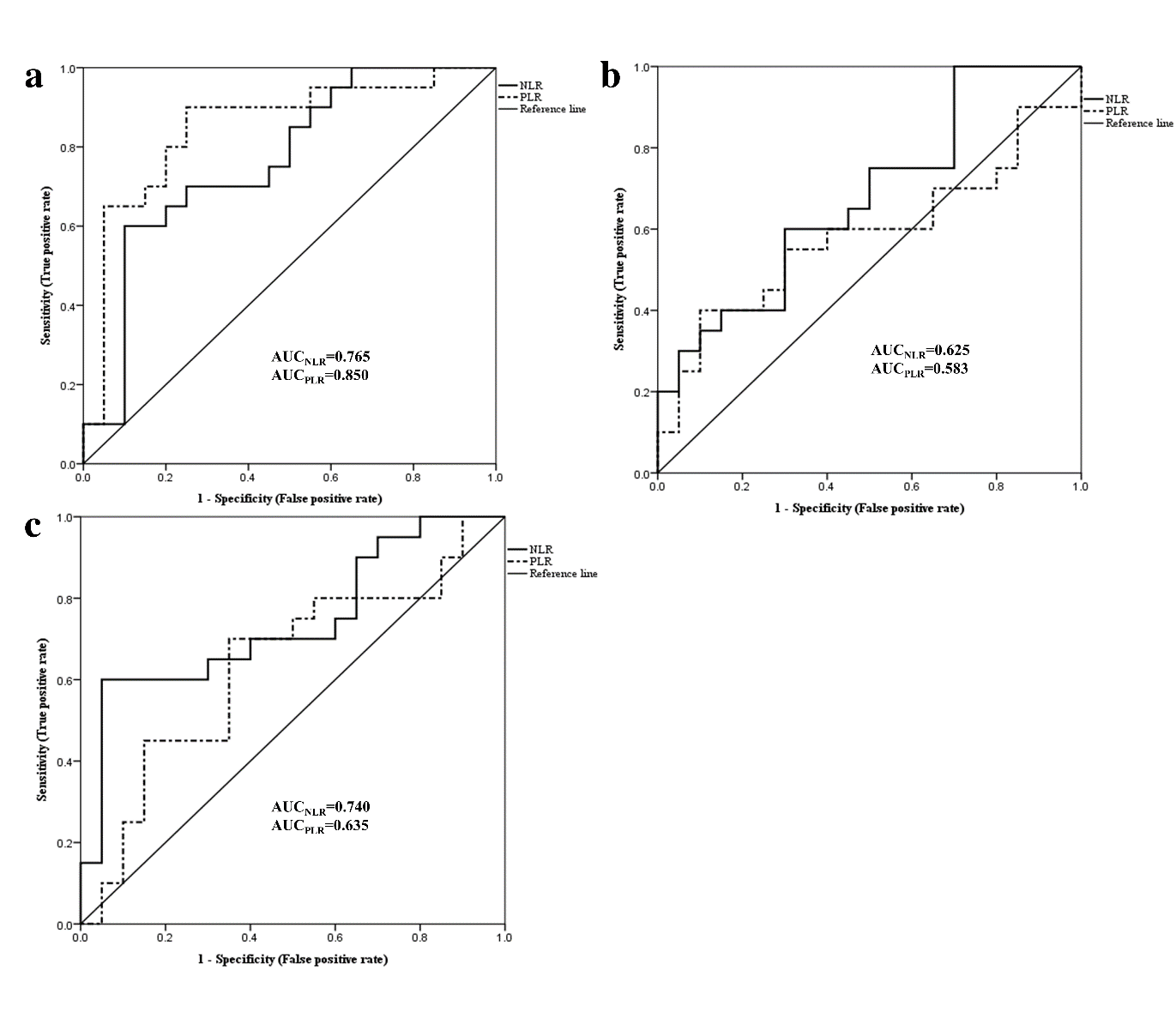
**Figure S6**. Receiver-operator characteristic curves of blood neutrophil-to-lymphocyte ratio (NLR) and blood platelet to-lymphocyte ratio (PLR) in dairy cows with low (LSCC) and middle somatic cell count (MSCC) at -4w (a), -3w (b), and -1w (c) relative to calving. N_(LSCC)_=23, N_(MSCC)_=20; AUC, area under the curve.


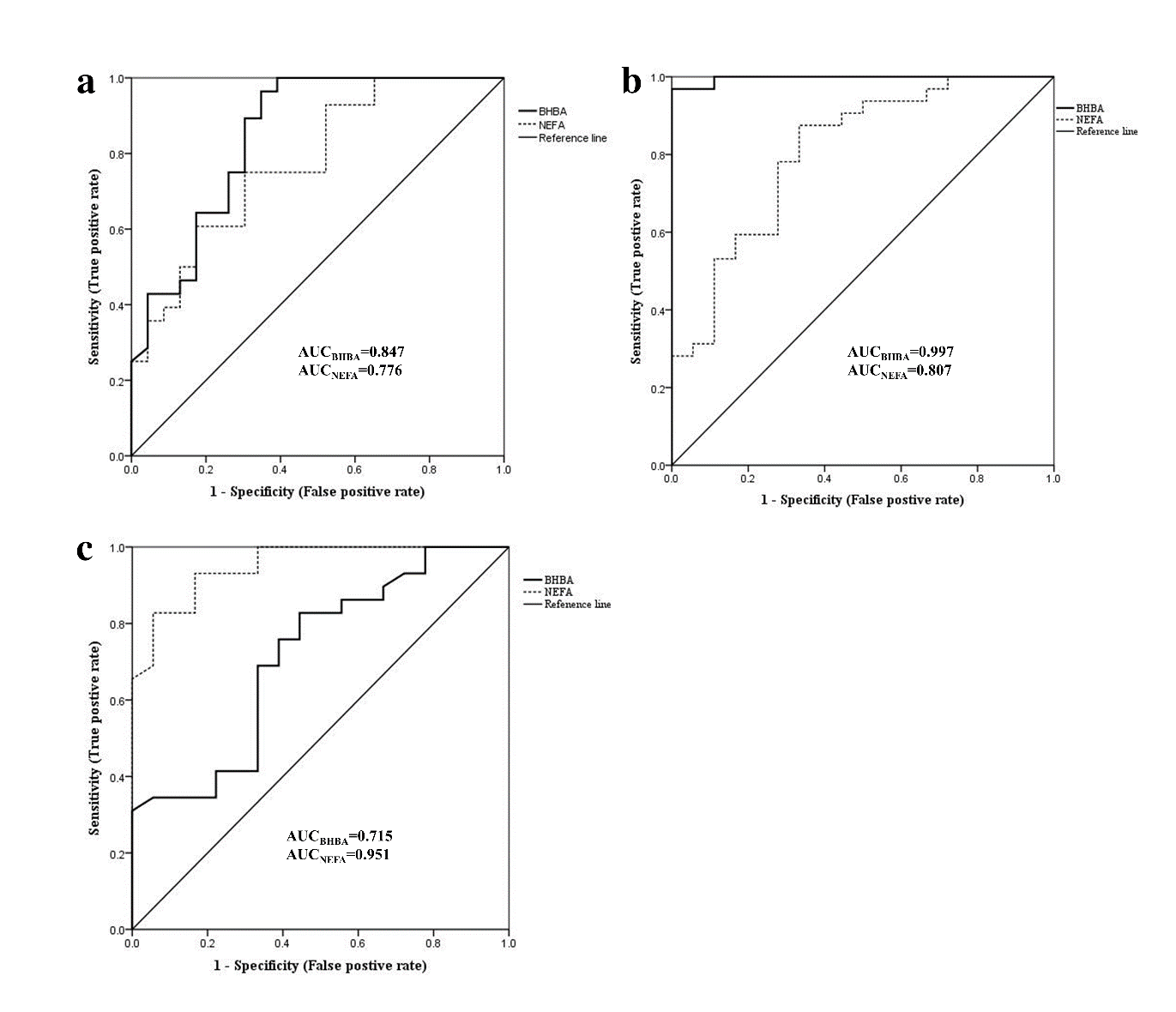


**Figure S7.** Receiver-operator characteristic curves of lipid metabolism analytes in dairy cows with low (LSCC) and high somatic cell count (HSCC) at -4w (a), -3w (b), and -1w (c) relative to calving. N_(LSCC)_=23, N_(HSCC)_=33; AUC, area under the curve; BHBA, β-hydroxybutyrate; NEFA, non-esterified fatty acid.


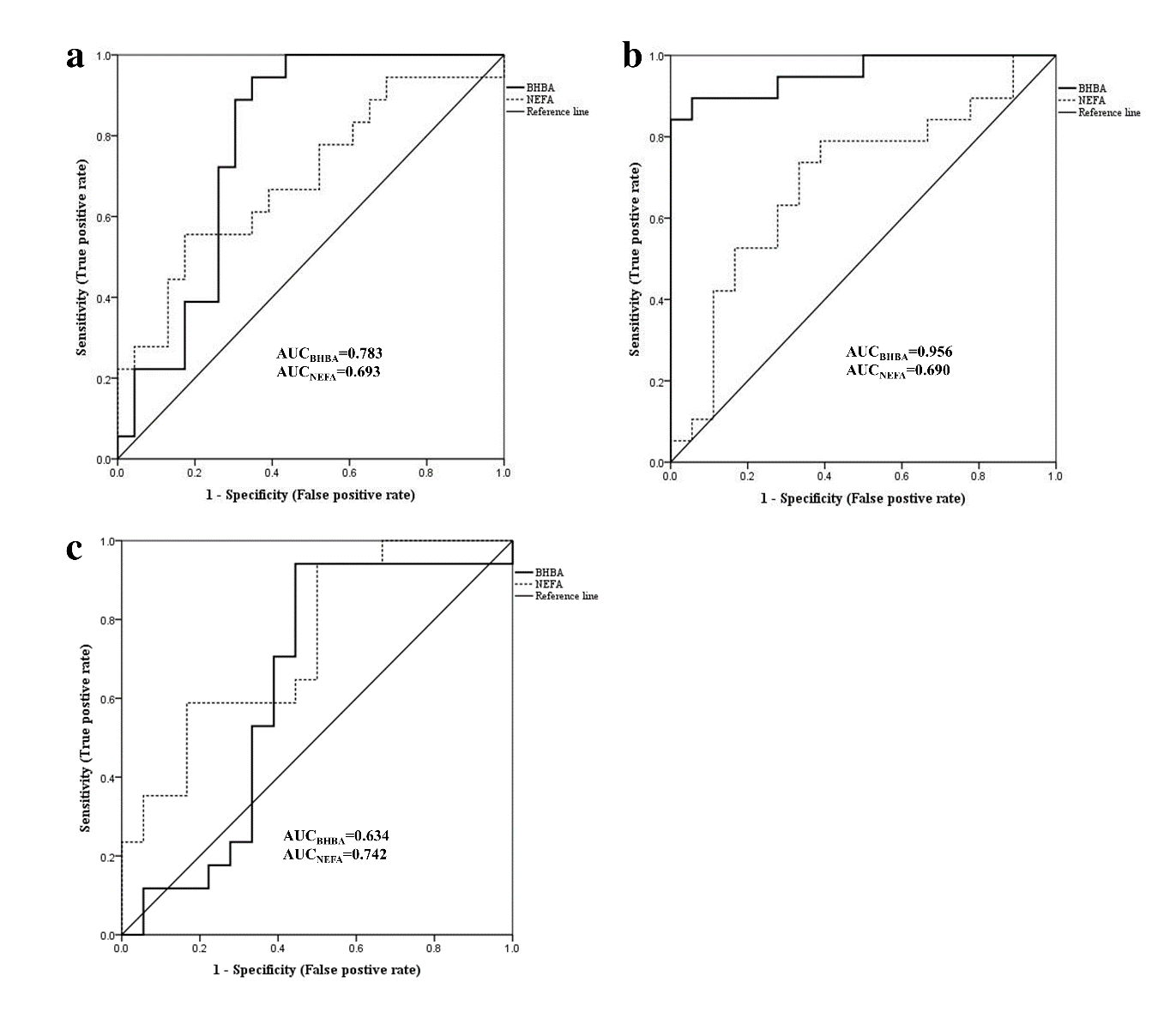


**Figure S8.** Receiver-operator characteristic curves of lipid metabolism analytes in dairy cows with low (LSCC) and middle somatic cell count (MSCC) at -4w (a), -3w (b), and -1w (c) relative to calving. N_(LSCC)_=23, N_(MSCC)_=20; AUC, area under the curve; BHBA, β-hydroxybutyrate; NEFA, non-esterified fatty acid.


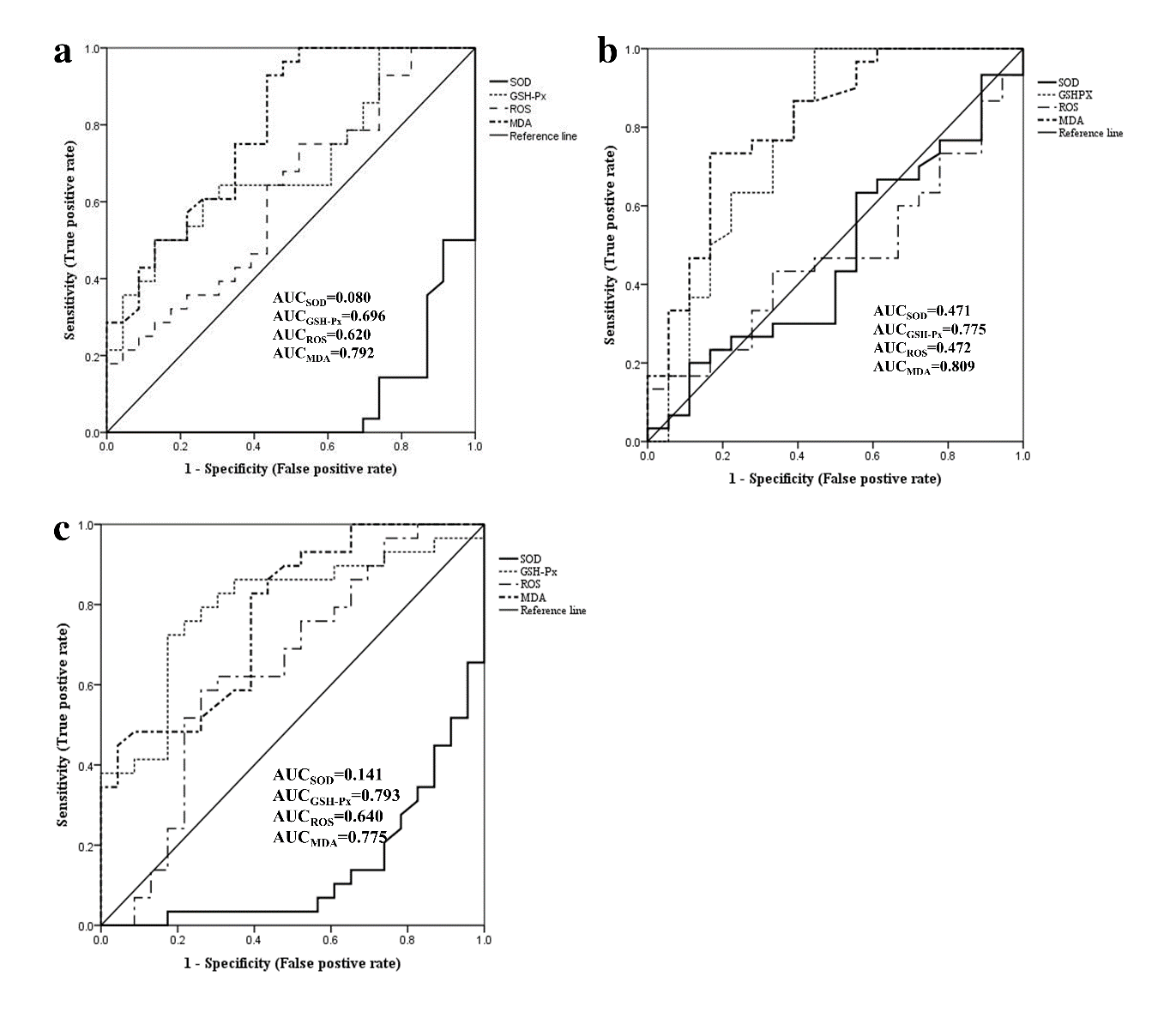


**Figure S9.** Receiver-operator characteristic curves of serum anti-oxidative analytes in dairy cows with low (LSCC) and high somatic cell count (HSCC) at -4w (a), -3w (b), and -1w (c) relative to calving. N_(LSCC)_=23, N_(HSCC)_=33; AUC, area under the curve; GSH-Px, glutathione peroxidase; MDA, malondialdehyde; ROS, reactive oxygen species (ROS); SOD, serum superoxide dismutase.

**
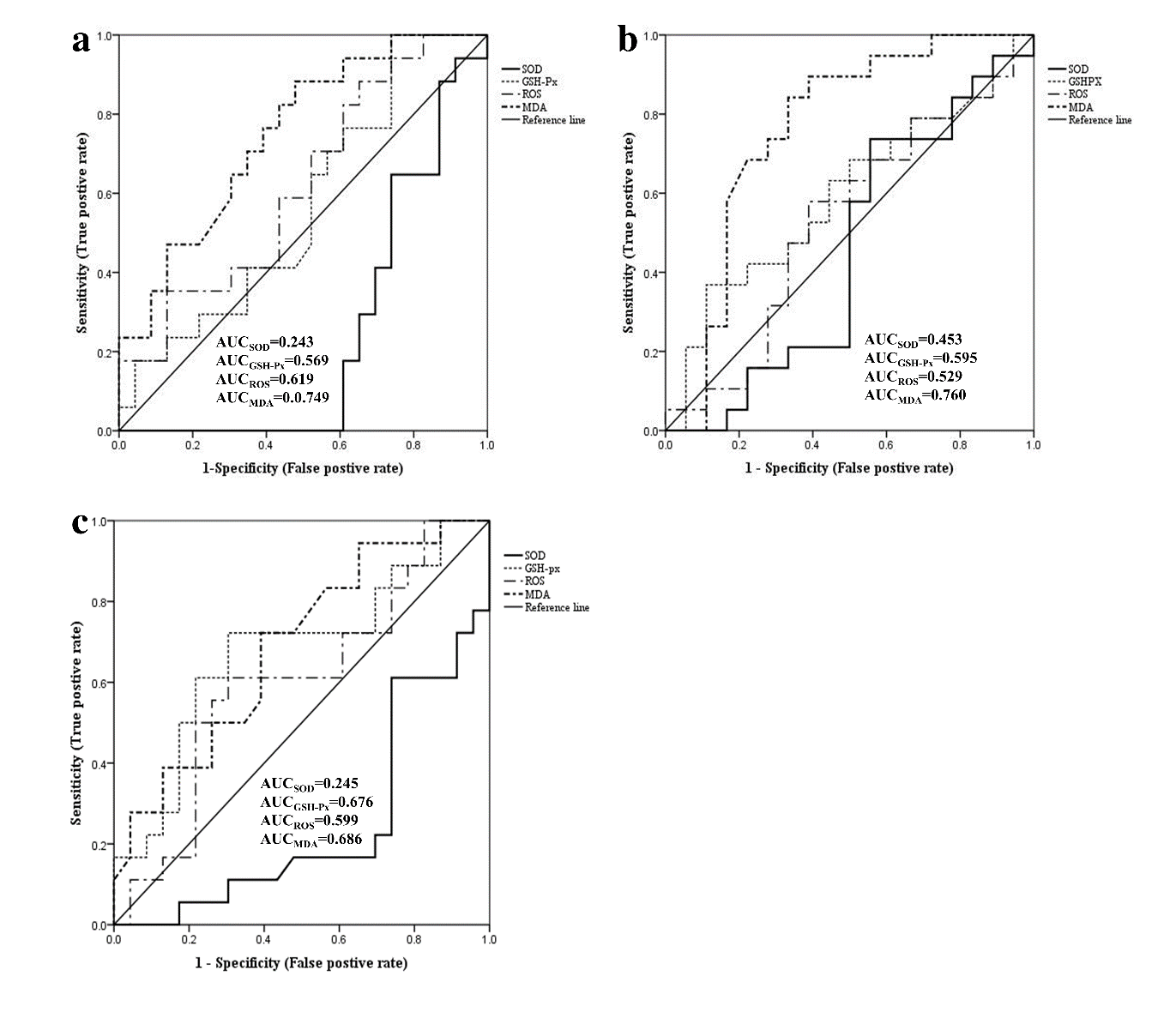
**

**Figure S10.** Receiver-operator characteristic curves of serum anti-oxidative analytes in dairy cows with low (LSCC) and middle somatic cell count (MSCC) at -4w (a), -3w (b), and -1w (c) relative to calving. N_(LSCC)_=23, N_(MSCC)_=20; AUC, area under the curve; GSH-Px, glutathione peroxidase; MDA, malondialdehyde; ROS, reactive oxygen species (ROS); SOD, serum superoxide dismutase.
